# Effectiveness of modified constraint-induced movement therapy for upper limb function intervention following stroke: A brief review

**DOI:** 10.1016/j.smhs.2021.08.001

**Published:** 2021-08-10

**Authors:** Manting Cao, Xia Li

**Affiliations:** The third affiliated hospital, Zhejiang Chinese Medical University, Hangzhou, China

**Keywords:** mCIMT, Stroke, Upper limb function

## Abstract

Neglecting the use of the affected limb in stroke patients can result in learned non-use. Modified constraint-induced movement therapy (mCIMT) is a form of rehabilitation therapy that limits the less paretic side, and through repeated and concentrated training improve the upper limb function of the paretic side. The aim of this paper is to develop a critical systematic review on the research evidence evaluating the effectiveness of applying mCIMT in the recovery of upper limb function in stroke patients. The outcome of this evaluation support that mCIMT significantly improves the upper limb function of stroke patients. Moreover, group mCIMT modality and TR (trunk restraint)+mCIMT modality provide greater benefits than mCIMT alone.

## List of abbreviations

ADL: Ability of Daily Life
BTX: A toxin
ARAT: Action Research Arm Test
AOU: Amount of Use
BTX-mCIMT: BTX injection combine with mCIMT
CR: Conventional rehabilitation
tDCS: Direct current stimulation
FMA: Fugel-Meyer Assessment
BTX-ICT: High-dose conventional therapy
MBI: Modified Barther Index
mCIMT: Modified constraint-induced movement therapy
MAL: Motor Activity Log
QOU: Quality of movement
tDCS: Transcranial direct current stimulation
TR: Trunk restraint
WFMT: Wolf Motor Function Test
BTX-mCIMT: BTX injection combine with mCIMT

## Introduction

Damage to upper limb function is one of the most common problems for stroke survivors.^1^ According to Singh and Pradhan,^2^ the upper limbs of the affected side of the stroke survivors have great motor dysfunction that seriously affect the quality of daily living. How to improve the function of upper limbs through effective rehabilitation measures is the key to helping stroke survivors improve their ability to complete daily living activities and independent activities.^3^ Modified constraint-induced movement therapy (mCIMT) is an effective treatment created to enhance upper limb function after a stroke^4^ and is one of the most important stroke rehabilitation measures.^5^ The main mechanism of mCIMT is to limit the less paretic side and through repeated and concentrated training improve the upper limb function of the paretic side.^6^ Morris et al.^7^defines mCIMT as having three principle parts: first, repeated training of impaired upper limbs for a few hours over 10 weekdays; second, a “transfer package” to guarantee that upper limb use in the patient's every day life; and finally, a limited use of the unharmed upper limb, forcing the individual to utilize the more impaired upper limb. Compared with traditional rehabilitation therapy, mCIMT significantly influenced motor control and daily activity function of the upper limbs.^8^

The aim of this study is to examine the rationality of choosing mCIMT as part of the treatment plan in restoring upper limb function in stroke patients.

## Method

### Literature search

In order to conduct a comprehensive search of the relevant data, 3 databases (Cochrane Library, PubMed and Pedro) were used. These three databases are reliable, and in order to conform to the guidelines of the comprehensive search and Boolean operators, the search terms were: stroke “AND” upper limb function “AND” modified constraint-induced movement therapy. Detailed inclusion and exclusion criteria are listed in Table 1. An Evidence-Based Analysis conducted by Medical Advisory Secretariat and Health Quality Ontario^9^ identified four relevant studies that investigated the effectiveness of CIMT in patents with upper limb dysfunction after a stroke. The results of this review found that moderate quality evidence exist providing support for the effectiveness of CIMT. However, only one study published in 2011, with limited results and restricted by small sample size. Thus, this topic deserves further investigation.

**Table 1:** Criteria for the literature search.

Inclusion criteria	Exclusion criteria
- Publish date:2011–2021. - Publish language: English. - Involved in both upper limb function of patients with stroke and modified constraint-induction movement therapy.	- Published before 2011 - Not yet published studies. - Not published in English. - Involved in stroke patients but did not specifically emphasize upper limb function. - The main intervention is not about modified constraint-induction movement therapy.

### Include studies

According to our literature search, only 7 studies conformed to the inclusion criteria and were used to assess the effectiveness of mCIMT therapy. Information for all 7 studies was listed in Table 2.

**Table 2:** Included studies.

Authors	Title	Participants
Yadav et al.^8^	Efficacy of Modified Constraint Induced Movement Therapy in the Treatment of Hemiparetic Upper Limb in Stroke Patients: A Randomized Controlled Trial	60 patients with previous stroke (during the period from October 2010 to April 2012). Modified Ashworth Scale: grade ≥3.
Bang et al.^6^	Effects of modified constraint-induced movement therapy combined with trunk restraint in chronic stroke: A double-blinded randomized controlled pilot trial	18 patients with previous stroke (more than 12 months) Modified Ashworth Scale: grade ​≥ ​3
Doussoulin et al.^10^	Effects of modified constraint-induced movement therapy in the recovery of upper extremity function affected by a stroke: a single-blind randomized parallel trial-comparing group versus individual intervention	36 patients with previous stroke. Modified Ashworth Scale: less than two point.
Singh and Pradhan^2^	Study to assess the effectiveness of modified constraint-induced movement therapy in stroke subjects: A randomized controlled trial	40 patients with subacute stroke (2–4 weeks after the onset); Modified Ashworth Scale: grade ≥1
Borch et al.^4^	Modified constraint-induced movement therapy early after stroke: Participants' experiences	3 patients with previous stroke (within 28 days)
Nasb et al.^11^	Comparison of the effects of modified constraint-induced movement therapy and intensive conventional therapy with a botulinum-a toxin injection on upper limb motor function recovery in patients with stroke	64 patients with stroke within one year; Modified Ashworth Scale: grade ≥1
Rocha et al.^12^	The impact of transcranial direct current stimulation (tDCS) combined with modified constraint-induced movement therapy (mCIMT) on upper limb function in chronic stroke: a double-blind randomized controlled trial	21 patients with stroke

### Critical analyses

6 randomized controlled trails and 1 qualitative analysis study were found in the literature search but only 5 studies that met the inclusion criteria. All studies have 2 common limitations: 1) Studies only focus on the short-term results of mCIMT. However, the stroke rehabilitation is a long-term process, and evaluating the long-term influence of this intervention for stroke survivors is important and has not received proper attention^13^; 2) As resources are a limitation, sample size of studies using stroke patients are usually small. Doussoulin et al.^10^ summarized their results and reported that extensive research supports the positive impact of mCIMT on upper limb function recovery, but most studies were limited by intervention time and resources. In 6 randomized controlled trials, 4 trials did not blind the investigator. Only Bang et al.^6^ and Rocha et al.^12^ used a double-blind approach. This study design is more effective in avoiding bias caused by the investigator in the implementation of treatment programs and assessments. Moreover, no patients follow-up after the intervention occurs as in the studies by Singh and Pradhan^2^ and Nasb et al.^11^ Without follow-up, little information is available for tracking the patients' subsequent development.

Yadav et al.^8^ performed a randomized controlled trial in stroke patients. A four-week mCIMT program in patients was compared to Conventional rehabilitation (CR) program and with CR program combined with mCIMT. CR group completed training 3 ​h per day for 4 weeks. Training included Ability of Daily Life (ADL) training, stretching, motor function and endurance training. CR+mCIMT program completed training upper limbs in daily functional tasks. The sample size of this study was small which likely impacted the accuracy of the results.^14^ However, Yadav et al.^8^ calculated the minimum sample size number required to detect effect size as 28 patients per group. This trial had 30 patients in each group and met the minimum requirements. Indeed, Yadav et al.^8^ used a randomized subject assignment to groups and clearly described the experimental design and analysis. Yadav et al.^8^ found the Fugel-Meyer Assessment (FMA) scores of mCIMT+CR group significantly higher than CR group's scores at 1 month (FMA1: *p*<0.0001, *es* = 0.2870) and 3 months (FMA3: *p*<0.0001, *es* = 0.4240). FMA is stroke-specific and can be used to determine the recovery of motor function after stroke and to evaluate the treatment effect.^15^ The FMA scores for the mCIMT+CR group reflects the importance of mCIMT training for improving abnormal motor patterns after stroke. mCIMT training can also help stroke survivors reverse the damage of upper limb and improved overall function.^8^ Yadav et al.^8^ also reported that the Amount of Use (AOU) scores and QOU (Quality of movement) scores on the Motor Activity Log (MAL) scale in mCIMT+CR group were significantly higher than CR group at 1 month and 3 months after intervention. This means that after the mCIMT intervention, stroke survivors have injured upper limbs increased activities of daily living both qualitatively and quantitatively.^8^ Yadav et al.^8^ provides scientific support that 4-week of mCIMT training is effective for restoring upper limb function in stroke patients.

Singh and Pradhan^2^ conducted a randomized controlled trial with the aim to understand the effectiveness of mCIMT in stroke patients. Stroke survivors were randomly assigned to an intervention group and a control group. Patients in the intervention group received 2 ​h of structured activity on the affected upper limb (5 times a week for 2 weeks). During the structured activities, participants wore mitts on the unaffected upper limbs. Control group patients received traditional physical therapy, and the unaffected upper limbs were not restricted. Their results showed that both Wolf Motor Function Test (WFMT) and FMA in the mCIMT group were significantly higher than the control group after intervention. No participant follow-up was completed. Follow-up is an important part of any clinical trial, because follow-up information on treatment outcomes can be collected while monitoring treatment effectiveness.^16^ Moreover, the sample size is most important with the work of Singh and Pradhan^2^ who had a smaller sample size than Yadav et al.^8^ These factors reduce study credibility. However, reasonable study design, clear structure, and randomized as part of the methods increase the reliability of the results.

Bang et al.^6^ conducted a trial to determine the effects of mCIMT combined with TR on upper limb function in stroke survivors. The use of TR is to reduce the compensatory effect of the trunk during training. Bang et al.^6^ randomized stroke patients to the TR+mCIMT group and to the mCIMT group. Patients in both groups experienced 20 sessions of intervention (1 ​h/day, 5 days/week for 4 weeks). Both groups were required to perform repeated task training with the injured upper limb while the unaffected upper limbs were confined with a mitt during training. The participants in the TR+mCIMT group's compensatory trunk movement was limited by elastic bandages. Bang et al.^6^ found that the scores for the Action Research Arm Test (ARAT), FMA, Modified Barther Index (MBI) in both groups were significantly improved after 4 weeks of intervention. Moreover, the mean values of TR+mCIMT group were higher than that of the mCIMT group. However, the relative smaller sample and low effect size reduce the strength of the study conclusions.

Considering the limitations of clinical resources and human resources, Doussoulin et al.^10^ conducted a randomized trail with stroke survivors to evaluate the clinical practicability of mCIMT. Randomized methods were used to assign participants to the intervention group (group modality, *n* ​= ​24) and the control group (individual modality, *n* ​= ​12). Both groups performed parallel interventions led by professionals 3 ​h per day for 10 days. Both types of mCIMT intervention significantly influenced restoration of the affected upper limbs function. Moreover, the benefits of group therapy remained 6 months after intervention. Doussoulin et al.^10^_,_ found that group mCIMT's intervention was most beneficial in optimizing treatment management and cost-effectiveness.

Borch et al.^4^ found that participants' satisfaction with mCIMT effectiveness is most important to understand.^4^ Daramilas and Jaspal^17^ found that patient satisfaction was based on the patients' experience and was an important indicator for evaluating the curative effect of any treatment. In order to gain insight into how mCIMT affected stroke patients, Borch et al.^4^ conducted in-depth interviews with 3 stroke patients who were required to receive mCIMT intervention 3 ​h per day for 2 weeks. The results of the analysis showed that after mCIMT treatment, the affected upper limb function of all 3 participants improved. Unfortunately, participants also stated they were still experiencing a lack of fine motor function.^4^ This study has 3 major limitations that may cause bias in study results. First, the sample size of Broch et al.^4^ study is extreme small, which reduces study credibility. Second, this study provided data concerning subjective feelings. The data was likely impacted by participants' connection to the mCIMT staff and researchers. Finally, these patients were treated with mCIMT along with other physical and occupational treatments. The possibility of other treatments affecting the results of the study can not be eliminated.

Nasb et al.^11^ examined whether A toxin (BTX) injection combine with mCIMT (BTX-mCIMT) showed better effectiveness than the BTX combine with high-dose conventional therapy (BTX-ICT) in stroke patients. The results support BTX-mCIMT therapy as having greater improvement of motor function and activities of daily living. However, when considering patient individual differences, the injection dose of BTX for each patient was not consistent. Moreover, the reliability of this study is limited by a small sample size and no follow-up.

Rocha et al.^12^ examined the effectiveness of transcranial direct current stimulation (tDCS) combined with mCIMT on upper limb function in stroke patients. Patients meeting the inclusion criteria were randomly assigned to Cathodal tDCS+mCIMT group, Anodal tDCS+mCIMT group, and a sham tDCS+mCIMT group. The results support that Anodal tDCS+mCIMT has significant higher effects than Cathodal tDCS+mCIMT on upper limb function in stroke patients, and both Athodal tDCS+mCIMT and Anodal tDCS+mCIMT are superior to sham tDCS+mCIMT treatment. However, the credibility of this study is limited by the small sample size.

## Discussion

This critical analysis supports previous findings that mCIMT in stroke patients is an effective therapy for restoring the function of affected upper limbs. However, no study has been completed that demonstrates the long-term effects of mCIMT on upper limb function in stroke patients. The question concerning the long-term effects of mCIMT is most certainly an area that need further evaluation.

4 studies reported the positive effectiveness of mCIMT as an intervention (Yadav et al.^8^; Singh and Pradhan^2^; Borch et al.^4^; Nasb et al.^11^). These studies demonstrated that mCIMT significantly improved the function of affected stroke patients’ upper limbs. In addition, Yadav et al.^8^ evaluated 4-week of mCIMT therapy and found this training was practical and clinical applicability for stroke patients. However, mCIMT improved the function of damaged limbs by countering compensation strategies.^5^ Bang et al.^6^ questioned whether the compensation strategy which relied on limiting the unaffected upper limbs was enough to optimize recovery. Reducing trunk compensation through trunk restriction is a strategy promoting recovery from stroke. Bang et al.^6^ demonstrated that the combination of TR and mCIMT was more effective than the use of mCIMT alone in the recovery of stroke survivors affected upper limb function. Stroke patients using only trunk compensation had a negative impact on recovery of motor function.^18^ Therefore, a reasonable hypothesis in need of testing is whether TR+mCIMT can enhance stroke recovery.

The argument against the use of group modality mCIMT is that patients do not receive targeted and individualized treatment. Logan et al.^19^ proposed that individualized treatments were more flexible and effective in dealing with potential and complex stroke patient problems. However, the benefits of group modality mCIMT reported by Doussoulin et al.^10^ were more convincing. Doussoulin et al.^10^ compared individual treatment with group mCIMT therapy and found that group mCIMT was more advantageous in providing improvement of the affected upper limbs. More importantly, this benefit lasted for 6 months after intervention. Several quality studies supported group-based exercise as having a positive impact on the mental health of people living in long-term care facilities while mobilizing patients' enthusiasm for exercise and improved patients' independence.^20^^,^^21^ Therefore, group mCIMT should become part of the management plan for recovery of stroke patients upper limb function. Worth noting that in the process of group rehabilitation, the role of the therapist should be a collaborator rather than an authority. Encourage patients to overcome difficulties and maintain self-confidence with empathy is most important.^21^

A recent finding by Rocha et al.^12^ is the association between mCIMT and brain stimulation is more effectiveness than mCIMT alone. Moreover, Anodal tDCS shows a stronger effect than Cathodal tDCS. Most quality studies recognize the effectiveness of tDCS for restoring upper limb function in stroke patients. As the downregulation of the activities of the normal motor cortex induced by normal upper limb restrain is intensified by Cathodal tDCS, the upregulation of the affected motor cortex activities are induced by the constraint-induced movement of upper limb of stroke effected side are intensified by Anodal tDCS. It is reasonable to combine mCIMT and tDCS to improve stroke patients’ upper limb function.^12^ However, existing evidence does not fully explain why Anodal tDCS has a better effect than Cathodal tDCS. Further examination of this problem is needed.

## Conclusion

Overall, this literature review examined the effectiveness of mCIMT in stroke patients. The outcome recommendation is to include mCIMT as part of the treatment plan in restoring upper limb function in stroke patients. Though few studies are available in this subject area, the key findings are that mCIMT therapy significantly improves stroke patients’ upper limb function. Moreover, group mCIMT modality, TR (trunk restraint)+mCIMT modality and mCIMT+tDCS have greater advantages over mCIMT alone.

## Submission statement

The manuscript has not been published and is not under consideration for publication elsewhere.

## Conflict of interest

The authors have no conflict of interest.

## Authors' contributions

M. Cao contributed to the conception of the study and wrote the manuscript. X. Li contributed to manuscript preparation.
